# miR-181a plays the tumor-suppressor role in chronic myeloid leukemia CD34^ +^ cells partially via SERPINE1

**DOI:** 10.1007/s00018-023-05036-8

**Published:** 2023-12-16

**Authors:** Xiuyan Zhang, Wenjuan Ma, Wen Xue, Yu Wang, Pan Chen, Quanxue Li, Yuan-Yuan Li, Xiaohui Hu, Yun Zhao, Haixia Zhou

**Affiliations:** 1 Cyrus Tang Medical Institute, Collaborative Innovation Center of Hematology, Soochow University, Suzhou, 215123 China; 2 The First Affiliated Hospital of Soochow University, Key Laboratory of Thrombosis and Hemostasis, Ministry of Health, Suzhou, 215006 China; 3 The Affiliated Nanhua Hospital, Department of Clinical Research Institute, Hengyang Medical School, University of South China, Hengyang, 421002 China; 4 Jianhu Country People’s Hospital, Yancheng, 224700 China; 5 Shanghai-MOST Key Laboratory of Health and Disease Genomics, Shanghai Institute for Biomedical and Pharmaceutical Technologies, Shanghai, 200237 China; 6 National Clinical Research Center for Hematologic Diseases, Suzhou, 215006 China; 7 MOE Engineering Center of Hematological Disease, Soochow University, Suzhou, 215123 China

**Keywords:** Chronic myeloid leukemia, miR-181a, SERPINE1, CD34^+^ cells, Imatinib mesylate

## Abstract

The formation of the *BCR-ABL* fusion gene drives human chronic myeloid leukemia (CML). The last 2 decades have witnessed that specific tyrosine kinase inhibitors (TKIs, e.g., imatinib mesylate, IM) against ABL1 improve disease treatment, although some patients still suffer from relapse and TKI resistance. Therefore, a better understanding of the molecular pathology of CML is still urgently needed. miR-181a-5p (miR-181a) acts as a tumor suppressor in CML; however, the molecular mechanism of miR-181a in CML stem/progenitor cells remains elusive. Herein, we showed that miR-181a inhibited the growth of CML CD34^+^ cells, including the quiescent subset, and sensitized them to IM treatment, while miR-181a inhibition by a sponge sequence collaborated with BCR-ABL to enhance the growth of normal CD34^+^ cells. Transcriptome data and biochemical analysis revealed that SERPINE1 was a bona fide and critical target of miR-181a, which deepened the understanding of the regulatory mechanism of SERPINE1. Genetic and pharmacological inhibition of SERPINE1 led to apoptosis mainly mediated by caspase-9 activation. The dual inhibition of SERPINE1 and BCR-ABL exhibited a significantly stronger inhibitory effect than a single agent. Taken together, this study demonstrates that a novel miR-181a/SERPINE1 axis modulates CML stem/progenitor cells, which likely provides an important approach to override TKI resistance.

**Supplementary Information:**

The online version contains supplementary material available at 10.1007/s00018-023-05036-8.

## Introduction

Chronic myeloid leukemia originates from hematopoietic stem cells acquiring the *BCR-ABL* fusion gene, which encodes a tyrosine kinase with consecutive activity. The development of tyrosine kinase inhibitors (TKIs, e.g., imatinib mesylate, IM) has witnessed the transition of CML from a fatal disease to a manageable ailment [1–3]. Nevertheless, up to 25% of patients in the chronic phase are confronted with drug resistance and relapse. For those who achieve long-term major molecular remission (MMR), the discontinuation of TKI results in frequent relapse [3]. Accumulating evidence indicates that CML stem cells are responsible for relapse and that the survival of these cells is not entirely dependent on BCR-ABL activity [4–9]. Therefore, the identification of other factors contributing to the survival of CML stem/progenitor cells will provide new clues to improve disease treatment.

microRNAs (miRNAs, or miRs) are 18 to 25-nucleotide long, non-coding single-stranded RNAs that regulate gene expression by degrading target transcripts or blocking their translation [10, 11]. It is well established that miRNAs play crucial roles in the growth and drug response of CML cells [12–14], including leukemic stem/progenitor cells [15–24]. Thus the study of miRNAs likely leads to the identification of novel predictive biomarkers or therapeutic targets [23, 25–27]. miR-181a-5p (hereinafter referred to as miR-181a) plays a tumor-suppressor role in CML [17, 28–32], and small activating (sa) RNAs targeting the promoter of miR-181a exhibits anti-leukemia activity through enhancing the expression of miR-181a [33]. We have reported the downregulation of miR-181a in CML CD34^+^ cells [17]; however, the direct target of miR-181a in these cells has not yet been elucidated.

Plasminogen activator inhibitor-1 (PAI-1) is one of the most important inhibitors of the plasminogen/plasmin system, which belongs to the conserved serine protease inhibitor (SERPIN) family and is designated as SERPIN family E member 1 (SERPINE1) [34, 35]. In general, SERPINE1 promotes tumor cell growth via its proangiogenic activity or antiapoptotic property [36–38]. A recent report shows that SERPINE1 facilitates the recruitment and polarization of macrophage in cancer, which provides a new mechanism explaining its pro-tumorigenic function [39]. SERPINE1 inhibitors exhibit antitumor activities in vitro and in vivo [40–44]. Recently, it has been reported that PAI-1 induced by TGF-β is responsible for retaining hematopoietic stem cells in the niche [45]. PAI-1 deficiency impairs BCR-ABL^+^ leukemic stem cells (LSCs), and a PAI-1 inhibitor (TM5614) plus IM shows a stronger effect to eradicate LSCs than a single agent in mice [46]. Importantly, TM5614 exhibits anti-leukemia activity in human CML cells in vitro [47]. Additionally, the combination therapy of TM5614 and TKIs is well tolerated in patients and achieves a higher molecular response (MR) rate than TKI treatment alone [48]. To date, the regulatory microRNA of SERPINE1 in human CML cells has not been reported and the effects of SERPINE1 silencing or SERPINE1 pharmacological inhibition on human CML stem/progenitor cells have not been well evaluated.

In the present study, we demonstrate that miR-181a plays an important role in the growth and TKI response of CML stem/progenitor cells. Moreover, SERPINE1 is identified as a bona fide target of miR-181a in CML cells. Genetic and pharmacological inhibition of SERPINE1 inhibits the in vitro and in vivo growth of BCR-ABL^+^ cells. Taken together, our data reveal a novel miR-181a/SERPINE1 axis modulating the growth and TKI response of CML stem/progenitor cells.

## Materials and methods

### Patients and cells

K562 cells, KU812 cells and 293 T cells were obtained from the Cell Bank of the Chinese Academy (www.cellbank.org.cn). K562 and KU812 cells were maintained in RPMI 1640 medium supplemented with 10% fetal bovine serum (FBS), and 293 T cells were maintained in DMEM medium plus 10% FBS. Murine BaF3 cells were maintained in RPMI 1640 medium supplemented with 10% FBS and 5 ng/mL mIL-3. BaF3/BCR-ABL cells were generated by *BCR-ABL* lentiviral transduction and maintained in RPMI 1640 medium plus 10% FBS. Bone marrow cells from human CML patients and healthy donors were obtained from the Hematological Biobank, Jiangsu Biobank of Clinical Resources, in accordance with the Declaration of Helsinki and with written informed consent approved by the Ethical Committee of Soochow University (SUDA20211224H03, Suzhou, China). A gradient centrifuge with Lymphocyte-H cell separation medium (Cedarlane Laboratories, Burlington, NC, USA) was used to obtain the nucleated cells, and an EasySep CD34 positive selection kit (STEMCELL Technologies, Vancouver, BC, Canada) was used to purify CD34^+^ cells. Quiescent CD34^+^ cells were isolated with Hoechst and Pyronin Y staining following Holyoake’s study [49]. The clinical characteristics of CML patients recruited in the present study are summarized in Supplementary Table S1.

### RNA extraction and RT-qPCR

For the assessment of mRNA, the extraction of total RNA and RT-qPCR analysis were performed as previously described [50]. For the measurement of microRNA, the total RNA was prepared by TRIzol (Thermo Scientific, Waltham, MA USA). 500 ng extracted RNA was reversed-transcribed into cDNA with a Transcriptor First Strand cDNA Synthesis Kit (Roche (China) Holding Ltd., Shanghai, China) and miRNA specific primers (GenePharma Co., Ltd., Shanghai, China) according to manufacturer’s protocol. Real-time quantification was carried out using 0.5 μL cDNA, 400 nM primers, 12.5 μL 2 × Power SYBR Green PCR Master Mix (ThermoFisher, Applied Biosystems, Foster City, CA, USA) on 7500 Real Time PCR System (Applied Biosystems). *β-ACTIN* or *u6* were used as endogenous controls, to normalize mRNA and miRNA input. The gene-specific primers are detailed in Supplementary Table S2.

### Lentiviral vectors and viral production

The shRNA sequences, mature miR-181a, and scramble sequence were synthesized and subcloned into a LV3 (H1/GFP&Puro) vector by GenePharma Co., Ltd. The sponge sequence against miR-181a were reported previously by others [51], and 3 consecutive sponge sequences were synthesized and subcoloned into LV3 vector by GenePharma Co., Ltd. All above sequences are shown in Supplementary Table S3. SERPINE1 cDNA was amplified with specific primers (Supplementary Table S2) and then subcloned into a lentiviral vector [52].

Lentiviral production was performed with polyethylenimine (PEI) transfection of 293 T cells. 6 × 10^6^ 293 T cells were plated into a  10 cm dish for 16 h and medium was replaced with fresh medium 2 h before transfection. 450 μL Opti-MEM containing 6 μg lentiviral vector and three packaging constructs was mixed with 50 μL PEI solution (1 mg/mL). After incubation at room temperature for 15 min, the mixture was added to 293 T cells. The lentiviruses were harvested 48 h and 72 h post transfection. The concentrated viruses were prepared by ultracentrifugation.

### Colony-forming cell assay

Normal bone marrow (NBM) and CML CD34^+^ cells were transduced with concentrated lentivirus, and the transduced CD34^+^ cells were isolated using fluorescence-activated cell sorting (FACS) (BD FACSAria III, Becton Dickinson, Franklin Lakes, NJ, USA). The colony-forming cell (CFC) assay was performed as previously described [52, 53]. A total of 1000 FACS-isolated cells were plated in methylcellulose medium (MethoCult H4230, STEMCELL Technologies) supplemented with a cocktail of cytokines, including stem cell factor (SCF, 50 ng/mL), interleukin-3 (IL-3, 20 ng/mL), interleukin-6 (IL-6, 20 ng/mL), granulocyte macrophage colony-stimulating factor (GM-CSF, 20 ng/mL), granulocyte colony-stimulating factor (G-CSF, 20 ng/mL), and erythropoietin (EPO, 3 IU/mL). The colonies were classified and counted 14–16 days later. For CFC assays with imatinib mesylate (IM), SCF and EPO were not supplemented.

### Western blotting

Protein samples were prepared using protein lysate buffer (Beyotime, Shanghai, China) and equal amounts of protein samples were separated with SDS-PAGE. These samples were transferred from the electrophoresed gel onto a polyvinylidene difluoride (PVDF) membrane (Millipore, Billerica, MA, USA). The membrane was subsequently incubated with specific antibodies and developed the films with an ECL detection system (GE Healthcare Life Sciences, Piscataway, NJ, USA). The antibodies used in this study are listed in Supplementary Table S4.

### Microarray analysis

K562 cells were infected with scramble and miR-181a viruses, and after 72 h GFP^+^ cells were sorted for RNA extraction. RNA was prepared using the RNeasy Mini Kit (QIAGEN, Hilden, Germany) following the manufacturer's recommendations. Microarray analysis was performed using Agilent whole human genome oligo-chips (4 × 180 K) in Shanghai Biotechnology Corporation and analyzed as previously described [50].

### Luciferase reporter assay

pEZX vector with the full-length 3′-UTR of SERPINE1 (NM_000602, from stop codon to the end, 1805 bp) and the blank control were purchased from GeneCopoeia, Inc. (Guangzhou, China, Cat#: HmiT054357-MT01). Two small constructs containing the predicted miR-181a recognition motifs (motif-1:1904–1926, motif-2:2899–2921) and their mutants (“ugaaugu” changed to “uacuagu”) were subcloned into pEZX blank vector between its *Eco*RI and *Xho*I sites using various primers (listed in Supplementary Table S2). The transfection was performed as previously described [53], 3 μg vector plasmid was transfected into 293 T cells using Lipofectamine 3000 (Thermo Scientific), or it was transfected into K562 cells using electrotransfection. 48 h later, the harvested cells were lysated to measure the luciferase and renilla activities using a Dual-luciferase Reporter Assay Kit (Promega, Madison, WI, USA) and presented as a Firefly/Renilla ratio. The relative reporter activities of individual samples in miR-181a-overexpressiong cells were normalized to control cells.

### Assays of ROS, mitochondrial activity and membrane potential

The cells were incubated with 10 µM 2′,7′-dichlorodihydrofluorescein diacetate (DCFH-DA) (Beyotime), and DMSO was added as the control. Then, the cells were analyzed by flow cytometry.

Mitochondrial activity was analyzed using MitoTracker Red (CMXRos) staining (Beyotime). The cells were stained with the dye and then analyzed by flow cytometry. The mean fluorescence intensity (MFI) of tiplaxtinin-treated cells was compared with untreated control cells.

Mitochondrial membrane potential was analyzed with a mitochondrial membrane potential detection kit (Beyotime). The cells were stained with JC-1 and were analyzed by flow cytometry. The fluorescent emission of JC-1 aggregates (590 nm, red) and that of JC-1 monomers (520 nm, green) were measured, and the ratio of the fluorescent intensities of aggregate to monomer dyes was determined.

### Activity assays of caspase-8 and caspase-9

Caspase-8 and 9 activities in cell lysates were determined using caspase-8 and 9 activity kits (Beyotime) according to the manufacture’s protocol. The lysate was mixed with corresponding substrate, incubated for 1 h at 37 °C, and measured OD405. The activities were normalized by the protein amount of the corresponding cell lysate, and expressed in units per microgram of protein.

### Animals

BaF3/BCR-ABL cell was used as a model to generate leukemia in mouse studies as previously described [53]. Six- to 8-week-old female BalB/C mice were lethally irradiated (650 cGy), and the sorted test or control cells (5 × 10^4^/mouse for miR-181a overexpression, 1 × 10^5^/mouse for Serpine1 silencing) combined with non-irradiated mouse bone marrow cells (5 × 10^5^/mouse) as donor cells were injected intravenously into the recipient mice (no less than 6 mice in each group). These mice were observed closely for signs of weight loss or lethargy. The diseased mice were dissected, and the spleens and livers were weighed. Cells from the spleen, liver, bone marrow, and peripheral blood were analyzed by flow cytometry to evaluate the infiltration of leukemic cells. All animals were maintained under pathogen-free conditions and in compliance with national and institutional guidelines. All protocols were approved by the Ethics Committee of Soochow University (SUDA20211224A01, Suzhou, China).

### Statistical analysis

All values are represented as the mean ± SEM from more than three biological replicates, and statistical analysis was performed with Student’s *t* test, in which *p* < 0.05 was considered significant. The Kaplan–Meier method was used to study the survival tendency, and the *P* value was estimated using the log-rank test.

## Results

### The expression of miR-181a is suppressed by BCR-ABL activity in CML stem/progenitor cells

**Fig. 1:**
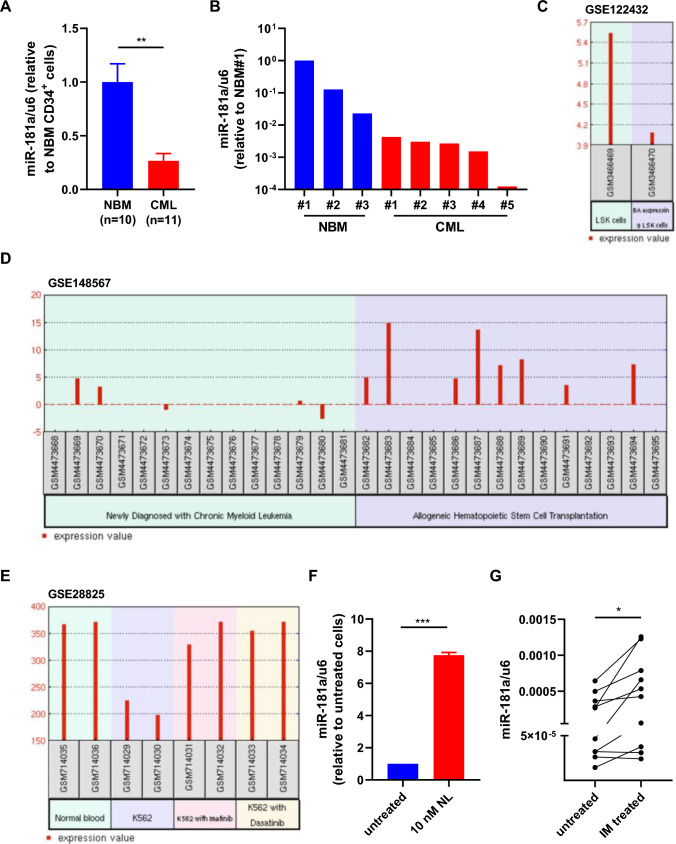
Aberrant expression of miR181a in CML stem/progenitor cells. **A** CD34^+^ cells were obtained from normal bone marrow (NBM) of healthy donors (n = 10) and chronic myeloid leukemia patients (n = 11) for RNA extraction, and the expression of miR-181a was measured by reverse transcription-quantitative polymerase chain reaction (RT-qPCR). **B** CD34^+^CD38^−^ cells were obtained from NBM (n = 3) and CML patients (n = 5) for RNA extraction, and the expression of miR-181a was measured by RT-qPCR. **C** The expression of miR-181a was inhibited by BCR-ABL in murine Lin^−^Sca1^+^c-Kit^+^ (LSK) cells (GSE122432). **D** The expression of miR-181a was increased in CML patients treated with allogenetic hematopoietic stem cell transplantation compared with newly diagnosed patients (GSE148567/GPL28379/154). **E** Treatment with imatinib and dasatinib enhanced the expression of miR-181a (GSE28825/has-miR-181a). **F** K562 cells were treated with nilotinib (NL), and the expression of miR-181a was analyzed by RT-qPCR in the control (untreated) and NL-treated cells. **G** CD34^+^ cells from CML patients (n = 8) were treated with imatinib mesylate (IM), and the expression of miR-181a was analyzed by RT-qPCR in the control (untreated) and IM-treated cells. Data are presented as the mean ± SEM from more than 3 biological replicates. Student’s *t* test was used to estimate the statistical significance. *p < 0.05, **p < 0.01, ***p < 0.001

To investigate the role of miR-181a in CML stem/progenitor cells, the expression of miR-181a in CML stem/progenitor cells compared with normal control cells was assessed by RT-qPCR in a cohort of patients recruited from the First Affiliated Hospital of Soochow University during 2018–2022. The results showed that miR-181a expression was significantly lower in CML CD34^+^ cells (n = 11) than in normal bone marrow (NBM) CD34^+^ cells (n = 10) (Fig. 1A). Moreover, miR-181a expression was lower in CML CD34^+^38^−^ cells than in their normal counterparts (Fig. 1B). In line with these data, miR-181a expression was decreased in BCR-ABL-expressing Lin^−^Sca-1^+^c-Kit^+^ (LSK) cells compared with control cells in the public database (GSE122432) (Fig. 1C); another set of data (GSE148567) showed that miR-181a expression was higher in CML patients underwent allogenetic transplantation than in newly diagnosed patients (Fig. 1D). To explore the possible relationship between miR-181a and BCR-ABL, we browsed the database to show that miR-181a expression was increased upon imatinib or dasatinib treatment in K562 cells compared with untreated cells (GSE28825) (Fig. 1E). Hence nilotinib (NL) was used to treat K562 cells, and the results showed that miR-181a expression was significantly enhanced (Fig. 1F). Similarly, imatinib treatment significantly increased miR-181a expression in CML CD34^+^ cells (n = 10) (Fig. 1G), though the extent was not as strong as that in K562 cells treated with NL.

### miR-181a acts as a tumor suppressor in CML stem/progenitor cells

**Fig. 2:**
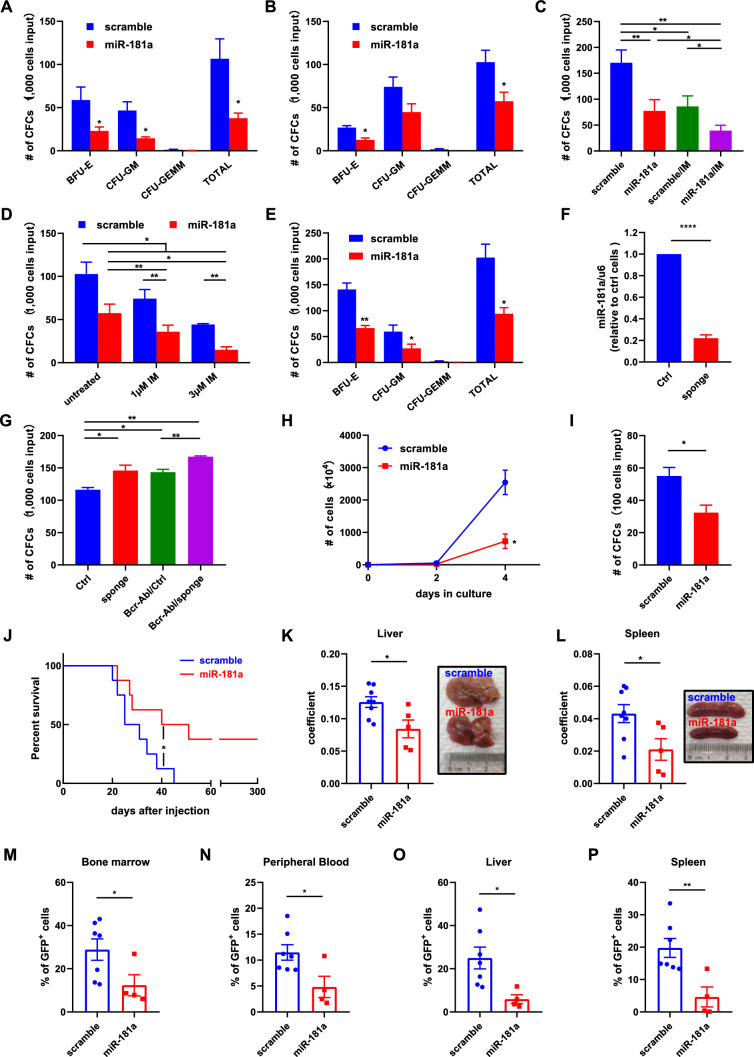
miR-181a inhibits the growth of CML cells and sensitizes them to imatinib mesylate treatment. **A** miR-181a and control (scramble) vectors were delivered into CML CD34^+^ cells, and the transduced CD34^+^ cells were purified by FACS and then assessed for their colony-forming cell (CFC) abilities. **B** Hoechst and Pyronin Y staining was used to isolate quiescent CD34^+^ cells. These cells were transfected with miR-181a and control (scramble) oligonucleotides and assayed for their CFC abilities. **C** miR-181a and scramble vector-transduced CML CD34^+^ cells were assayed for their CFC abilities with or without imatinib mesylate (IM). **D** miR-181a and scramble oligonucleotide-transfected quiescent CML CD34^+^ cells were plated for the CFC assay with or without IM. **E** miR-181a and control (scramble) vectors were delivered into normal bone marrow (NBM) CD34^+^ cells from healthy donors, and the transduced CD34^+^ cells were purified by FACS and then assessed for their CFC abilities. **F** A sponge against miR-181a and control (Ctrl) vectors were delivered into NBM CD34^+^ cells, the transduced CD34^+^ cells were purified by FACS and miR-181a expression was measured by RT-qPCR. **G** NBM CD34^+^ cells were transduced with various viral vectors as indicated, and the FACS purified cells were assayed for their CFC abilities. **H, I** miR-181a and control vectors were delivered into BaF3/BCR-ABL cells, the transduced cells were purified by FACS and then the growth (H) and CFC production (I) of these cells were assessed. **J** miR-181a-transduced BaF3/BCR-ABL cells and control cells (5 × 10^4^ cells/mouse) were injected into lethally irradiated mice through the tail vein (8 mice in each group). Kaplan–Meier method was used to study the survival of each group of mice. **K, L** The diseased mice from the miR-181a and scramble groups were dissected, and the liver and spleen were weighed. The coefficients of the liver (K) and spleen (L) of these two groups were compared. Representative photos of these organs are shown. **M–P** Leukemic cells (GFP^+^) from the miR-181a and scramble groups were analyzed by flow cytometry. The percentage of GFP^+^ cells in the bone marrow (M), peripheral blood (N), liver (O), and spleen (P) was compared between these two groups. Data are presented as the mean ± SEM from more than 3 biological replicates. Student’s *t* test was used to estimate the statistical significance. *p < 0.05, **p < 0.01

To study the functional role of miR-181a in leukemic stem/progenitor cells, miR-181a and scramble sequences were delivered into CML CD34^+^ cells by lentiviral vectors, and then the CFC production of the transduced CD34^+^ cells was assayed. The data showed that the CFC production of miR-181a-transduced cells was significantly lower than that of the control cells (Fig. 2A). Quiescent CML CD34^+^ cells were obtained and transfected with miR-181a and scramble oligonucleotides, and the results showed that miR-181a significantly suppressed the CFC production of quiescent CD34^+^ cells compared with the control (scramble) (Fig. 2B). miR-181a-transduced and control CML CD34^+^ cells were subjected to IM treatment, and the data showed that miR-181a enhanced the IM response of CML CD34^+^ cells (Fig. 2C). Similarly, miR-181a rendered quiescent CML CD34^+^ cells more sensitive to IM treatment (Fig. 2D).

Next, the function of miR-181a in NBM CD34^+^ cells was studied. miR-181a overexpression in NBM CD34^+^ significantly suppressed CFC production of these cells compared with the control (Fig. 2E). A sponge sequence, which entraps miR-181a was introduced to successfully inhibit miR-181a expression in NBM CD34^+^ cells (Fig. 2F). The sponge and BCR-ABL significantly enhanced the CFC production of NBM CD34^+^ cells compared with the control; moreover, the sponge collaborated with BCR-ABL to yield significantly more CFC production than the sponge or BCR-ABL alone (Fig. 2G).

Although the tumor-suppressor role of miR-181a in CML cells has been reported [17, 28–32], the effect of miR-181a on BCR-ABL-induced leukemogenesis has not been studied. Herein, we showed that miR-181a overexpression significantly suppressed the growth and CFC production of BaF3/BCR-ABL cells (Fig. 2H, I). In animal studies, Kaplan–Meier analysis indicated that miR-181a significantly delayed BCR-ABL-induced leukemia (Fig. 2J). The coefficients of both the liver and the spleen were significantly lower in the miR-181a-overexpressing group than in the control group (Fig. 2K, L). The infiltration of leukemic cells in the bone marrow, peripheral blood, liver, and spleen of the diseased mice in the miR-181a-overexpressing group was significantly lower than in the control group (Fig. 2M–P). Overall, these data demonstrated that miR-181a played a tumor-suppressor role in CML stem/progenitor cells.

### SERPINE1 is a bona fide target of miR-181a in CML CD34^+^ cells

**Fig. 3:**
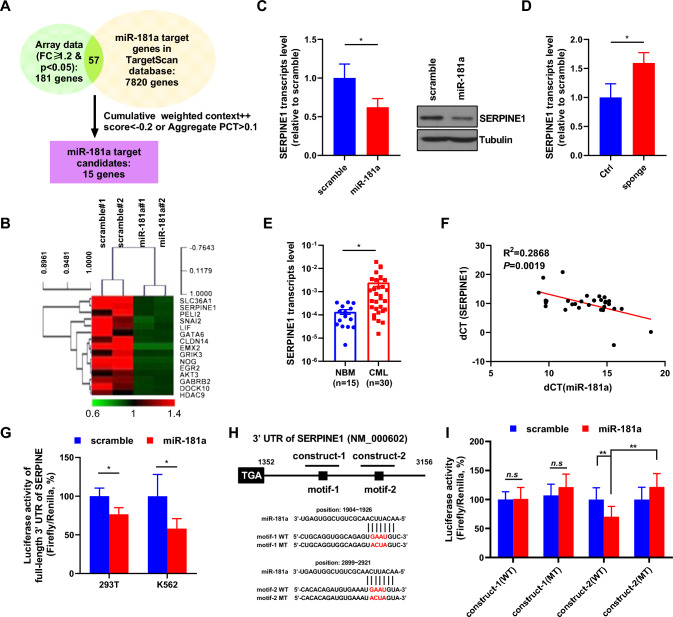
miR-181a directly regulates the expression of SERPINE1 in CML cells. **A** Venn diagram shows how the miR-181a targets were screened in this study. Transcriptome analysis was performed to compare miR-181a-transduced and control (scramble) K562 cells. A total of 181 genes were downregulated upon miR-181a expression. The prediction by the TargetScan database indicated that over 7000 genes are putative targets of miR-181a. The intersection of these two screens contained 57 candidate genes, and 15 candidates were yielded with more stringent criteria. **B** These 15 miR-181a candidate target genes are displayed in a heatmap comparing miR-181a-transduced and control cells. **C** miR-181a-transduced and control K562 cells were obtained for RNA preparation, and the transcript expression of SERPINE1 was measured by RT-qPCR. The expression of SERPINE1 was also analyzed by Western blotting. **D** A sponge against miR-181a and control (Ctrl) vectors were delivered into normal bone marrow (NBM) CD34^+^ cells from healthy donors, and the transcript expression of SERPINE1 was measured by RT-qPCR. **E** NBM CD34^+^ cells from healthy donors (n = 15) and CD34^+^ cells from CML patients (n = 30) were collected for RNA preparation. Then, the transcript expression of SERPINE1 was measured by RT-qPCR. **F** The correlation between miR-181a expression and SERPINE1 expression was analyzed in CML CD34^+^ cells. **G** A reporter vector containing the full-length 3′-UTR of SERPINE1 was transfected into miR-181a-transduced and control 293 T cells or K562 cells. The reporter activities were measured. **H** Two miR-181a recognition motifs were predicted in the 3′-UTR of SERPINE1. The sequences of these motifs and their mutants are shown together with the positions in SERPINE1 transcript. Constructs containing wild-type (WT) motif 1, mutant (MT) motif 1, WT motif 2, and mutant motif 2 were subcloned into reporter vectors. **I** The activities of these vectors in miR-181a-transduced and control K562 cells were measured. Data are presented as the mean ± SEM from more than 3 biological replicates. Student’s *t* test was used to estimate the statistical significance. *p < 0.05, **p < 0.01

Identifying the direct target of miR-181a is the key to understanding how miR-181a regulates the growth and TKI response of CML stem/progenitor cells. Microarray data were generated to compare miR-181a-transduced K562 cells with control cells. A total of 181 transcripts were downregulated upon miR-181a. In silicon analysis showed that over 7 thousand transcripts are potential miR-181a direct targets in the TargetScan database. The intersection of downregulated transcripts upon miR-181a overexpression and the potential direct targets included 57 transcripts. When more stringent criteria were used, the candidate list was shortened to 15 transcripts (Fig. 3A), whose expression in miR-181a-transduced and control K562 cells was displayed in a heatmap (Fig. 3B). Among these candidates, GATA6 is a reported miR-181a target [54]. RT-qPCR analysis showed that GATA6 expression was significantly decreased in miR-181a-transduced cells than in control cells (Fig. S1), which supported the validity of our microarray data. SERPINE1 was chosen as it promotes cancer cell growth and its inhibitors are available. Hence, we performed studies to validate that both the transcript and protein expression of SERPINE1 was decreased in miR-181a-transduced cells compared with control cells (Fig. 3C). Conversely, the sponge against miR-181a significantly enhanced SERPINE1 expression in NBM CD34^+^ cells (Fig. 3D). Interestingly, SERPINE1 expression was significantly higher in CML CD34^+^ cells than in NBM CD34^+^ cells (28-fold, *p* < 0.05) (Fig. 3E), and SERPINE1 expression was significantly correlated with miR-181a expression in CML CD34^+^ cells (Fig. 3F).

A reporter vector containing the full-length 3′-UTR of SERPINE1 was delivered into miR-181a-overexpressing and control 293 T or K562 cells, and the results showed that miR-181a significantly decreased reporter activity compared with the control (Fig. 3G). Two potential miR-181a recognition motifs were predicted (Fig. 3H). Two constructs (~ 200 bp) containing the predicted motifs (wild-type, WT) and their mutants (MT), were subcloned into the reporter vector. The reporter assay in K562 cells showed that the reporter activity of construct-2 [containing motif-2 (2899–2921)] was significantly suppressed by miR-181a but not construct-1 [containing motif-1 (1904–1926)], and the construct-2 mutant was not regulated by miR-181a (Fig. 3I). Therefore, miR-181a directly regulates the expression of SERPINE1 in CML stem/progenitor cells through a specific recognition motif.

### SERPINE1 silencing inhibits the growth of CML stem/progenitor cells and sensitizes them to IM treatment

**Fig. 4:**
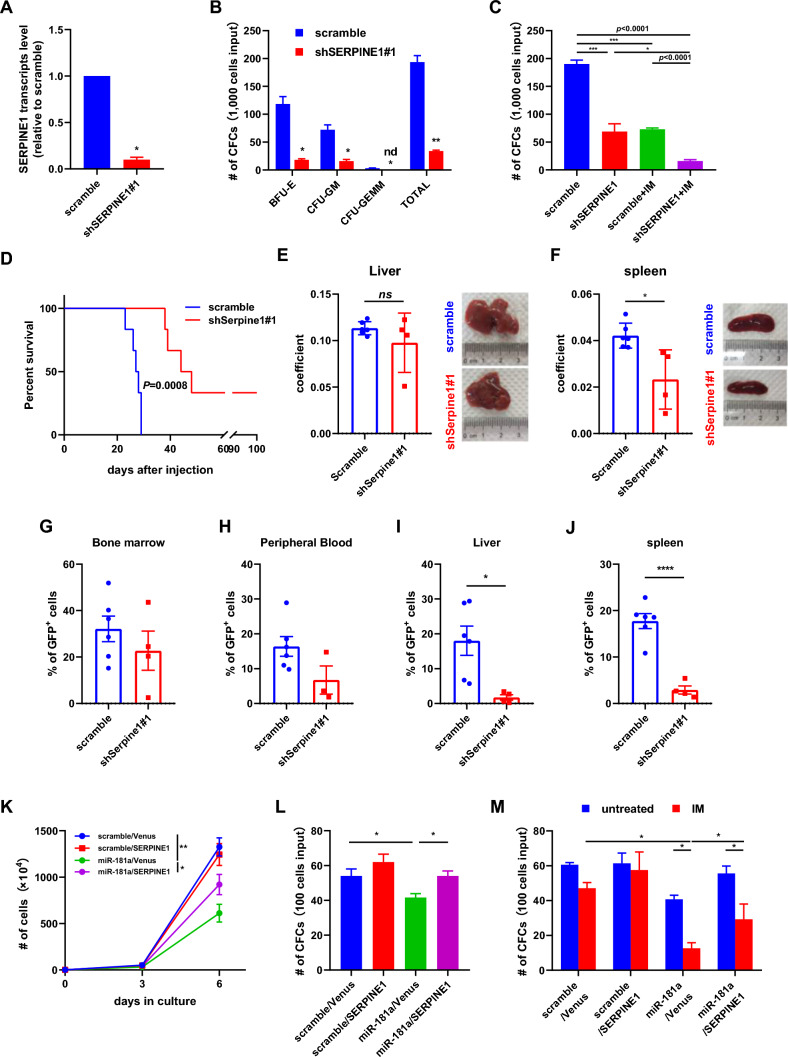
Inhibition of SERPINE1 suppresses the growth of CML cells and sensitizes them to imatinib mesylate treatment. **A–C** shSERPINE1#1 and control (scramble) vectors were delivered into CML CD34^+^ cells, and the transduced CD34^+^ cells were purified by FACS. Then, the expression of SERPINE1 in these cells was assessed by RT-qPCR (A), the colony-forming cell (CFC) abilities of these cells were measured (B), and the response of these cells to imatinib mesylate (IM) treatment was also assessed (C). **D** shSerpine1-transduced BaF3/BCR-ABL cells and control cells (5 × 10^4^ cells/mouse) were injected into lethally irradiated mice through the tail vein (6 mice in each group). The survival of these mice was studied with Kaplan–Meier method. **E, F** The diseased mice from the shSerpine1 and scramble groups were dissected, and the liver and spleen were weighed. The coefficient of liver (E) and the coefficient of spleen (F) of these two groups were compared. Representative photos of these organs are displayed as well. **G–J** Leukemic cells (GFP^+^) from the shSerpine1 and scramble groups were analyzed by flow cytometry. The percentage of GFP^+^ cells in the bone marrow (**G**), peripheral blood (H), liver (I), and spleen (J) was compared between these two groups. **K–M** A lentiviral vector to overexpress SERPINE1 was constructed. K562 cells were transduced with various lentiviral vectors as indicated. The growth (K), CFC abilities (L), and IM response (M) of variously transduced cells were measured. These cells included scramble/Ctrl, scramble/SERPINE1, miR-181a/Ctrl, and miR-181/SERPINE1. Data are presented as the mean ± SEM from more than 3 biological replicates. Student *t* test was used to estimate the statistical significance. *p < 0.05, **p < 0.01, ***p < 0.001, ****p < 0.0001

To study the function of SERPINE1 in CML cells, two independent shRNA and control (scramble) sequences were delivered into K562 cells, and RT-qPCR and Western blotting showed that these two shRNAs effectively inhibited SERPINE1 expression (Fig. S2A, B). SERPINE1 silencing inhibited the growth and CFC production of K562 cells (Fig. S2C, D). As these two shRNA sequences displayed similar inhibitory effect, we conduced following studies with only one of them (shSERPINE1#1). The result showed that SERPINE1 silencing rendered K562 cells more sensitive to IM treatment (Fig. S2E). RT-qPCR showed that SERPINE1 expression in CML CD34^+^ cells was significantly suppressed by shSERPINE1#1 (Fig. 4A), which led to significant reduction of CFC production of CML CD34^+^ cells and hypersensitivity of these cell upon IM treatment (Fig. 4B, C). It was noted that SERPINE1 silencing also significantly suppressed the CFC production of normal CD34^+^ cells from healthy donors (Fig. S2E).

To examine the effect of Serpine1 silencing on BCR-ABL-induced leukemogenesis, two independent shRNA sequences against Serpine1 and the control (scramble) were delivered into BaF3/BCR-ABL cells, and both effectively inhibited Serpine1 expression (Fig. S3G). Serpine1 silencing significantly suppressed the growth and CFC production of BaF3/BCR-ABL cells (Fig. S2H, I). As these two shRNAs had similar inhibitory effects, we chose one (shSerpine1#1) to conduct animal studies. Kaplan–Meier analysis showed that Serpine1 silencing significantly inhibited BCR-ABL-induced leukemia (Fig. 4D). The coefficient of the spleen but not the liver was significantly decreased by Serpine1 silencing (Fig. 4E, F). Moreover, Serpine1 silencing led to significantly fewer leukemic cells in both the liver and spleen, as analyzed by flow cytometry (Fig. 4G–J).

Finally, a rescue experiment was performed to elucidate the role of SERPINE1 in miR-181a-mediated regulation of the growth and IM response of CML cells. The results showed that SERPINE1 overexpression did not enhance the growth of K562 cells or confer IM resistance to these cells; however, this action partially rescued the growth inhibition and IM hypersensitivity induced by miR-181a (Fig. 4K–M). Overall, this study identified a novel miR-181a/SERPINE1 axis that modulates the growth and TKI response of CML cells.

### Tiplaxtinin and IM collaboratively inhibit the growth of CML stem/progenitor cells

**Fig. 5:**
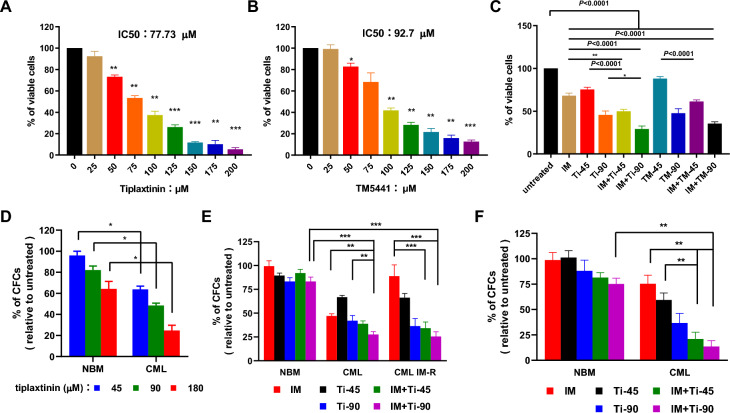
Compound against SERPINE1 inhibits the in vitro growth of CML stem/progenitor cells.** A–C** Two compounds against SERPINE1 were used in the present study. The inhibitory effect of tiplaxtinin (A) and TM5441 (B) was assessed by CCK-8 methods with K562 cells. Then, the viability of K562 cells (C) treated with the combination of imatinib mesylate (IM) and SERPINE1 inhibitor was also studied. **D** The effect of tiplaxtinin on colony-forming cell (CFC) production of normal bone marrow (NBM) and CML CD34^+^ cells was measured. **E** The effect of the combination of tiplaxtinin and IM on the CFC production of NBM and CML CD34^+^ cells was analyzed. The CML CD34^+^ cells were divided into two groups: the IM-sensitive group (survival of CFC production less than 60% in the presence of IM at 5 μM) and the IM-resistant group (survival of CFC production more than 60% in the presence of IM at 5 μM). **F** Quiescent CML CD34^+^ cells were purified with Hoechst and Pyronin Y staining method, and then the effect of the combination of tiplaxtinin and IM was analyzed. Quiescent NBM CD34^+^ cells were used as controls in this study. Data are presented as the mean ± SEM from more than 3 biological replicates. Student’s *t* test was used to estimate the statistical significance. *p < 0.05, **p < 0.01, ***p < 0.001

SERPINE1 inhibitors exhibit anti-leukemia activity [47]; however, their effect on CML stem/progenitor cells has not yet been reported. Herein, two types of SERPINE1 inhibitors (tiplaxtinin and TM5441) were studied (Fig. S3A). The effects of these two inhibitors on the viability of K562 cells, KU812 cells and BaF3/BCR-ABL cells were analyzed (Fig. 5A, B, Fig. S3B, S3C, Fig. S4A). The results showed that both had inhibitory effects on the viability of CML cells and that CML cells were more sensitive to tiplaxtinin treatment than TM5441 treatment. The effect of tiplaxtinin on the growth and CFC production of K562 cells was also analyzed (Fig. S3D, E). Next, the effect of the combination of the SERPINE1 inhibitor and IM was studied, and both tiplaxtinin and TM5441 collaborated with IM to inhibit the viability of K562 cells and KU812 cells (Fig. 5C, Fig. S3F). The combination of tiplaxtinin and IM showed a stronger inhibitory effect than the combination of TM5441 and IM. Additionally, the combination of tiplaxtinin and IM showed a stronger inhibitory effect on BaF3/BCR-ABL cells than a single agent (Fig. S4B). Then, tiplaxtinin was chosen for the following studies. The results showed that tiplaxtinin inhibited the CFC production of CML CD34^+^ cells in a dose-dependent manner and exhibited a stronger inhibitory effect on leukemic cells than on normal control cells (Fig. 5D). The effect of the combination of tiplaxtinin and IM on the CFC production of NBM CD34^+^ cells, IM-sensitive CML CD34^+^ cells, and IM-resistant CML CD34^+^ cells was studied. In this assay, CML CD34^+^ cells were divided into two categories based on CFC survival measurement in the presence of 5 μM IM following the criteria of Jiang’s study [55]. The individual CML sample with a survival rate less than 60% was assigned as IM-sensitive; otherwise, the sample was IM-resistant. The combination treatment had a stronger inhibitory effect on both IM-sensitive and IM-resistant CML CD34^+^ cells than on NBM CD34^+^ cells (Fig. 5E), and effectively induced apoptosis in CML CD34^+^ cells (Fig. S5). Finally, the combination treatment manifested a much stronger inhibitory effect on leukemic quiescent CD34^+^ cells than on normal quiescent CD34^+^ cells (Fig. 5F).

### Tiplaxtinin, a SERPINE1 inhibitor, activates caspase-9 to induce apoptosis of CML cells

**Fig. 6:**
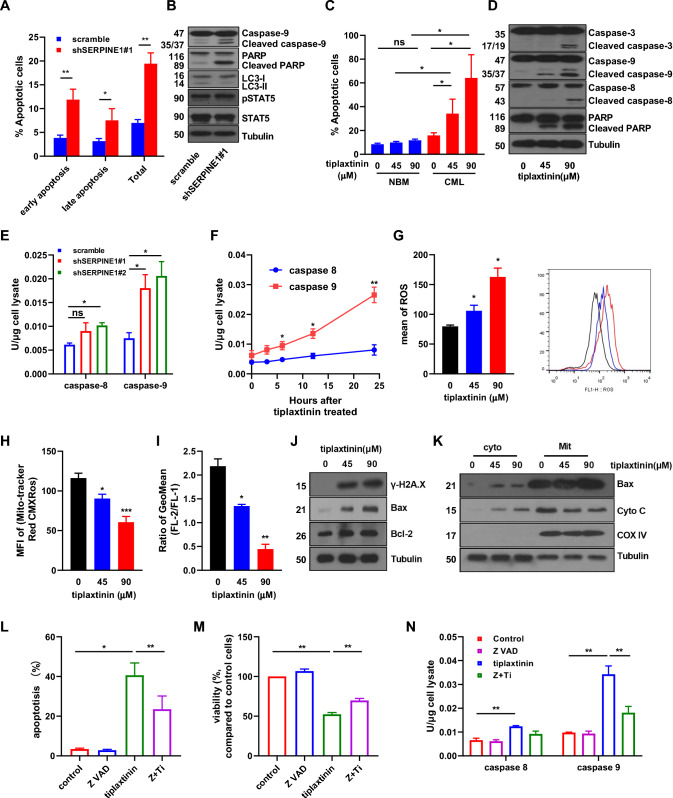
Tiplaxtinin induces apoptosis of CML cells mainly through caspase-9 activation. **A, B** shSERPINE1 transduced and control (scramble) K562 cells were analyzed by Annexin-V/propidium iodide (PI) staining for apoptotic cells, and the results were summarized statistically (A). These cells were also analyzed by Western blotting (B). **C** Normal bone marrow (NBM) CD34^+^ cells and CML CD34^+^ cells were treated with tiplaxtinin (45 and 90 μM). The treated cells and their untreated control cells were analyzed by Annexin-V/PI staining, and the apoptotic cells were summarized statistically. **D** Tiplaxtinin-treated K562 cells and untreated control cells were analyzed by Western blotting. **E** SERPINE1-silenced and control K562 cells were obtained, and the activities of caspase-8 and caspase-9 were assessed. **F** K562 cells were treated with tiplaxtinin, and the cells were collected at various time points and subjected to the measurements of caspase-8 and caspase-9 activities. **G** K562 cells were treated with tiplaxtinin (45 and 90 μM), and the reactive oxygen species (ROS) of the treated and control cells were measured and compared (left panel). The representative flow cytometry profile is displayed (right panel). **H, I** The active mitochondria (H) and membrane potential (I) of tiplaxtinin-treated (45 and 90 μM) cells and control cells were measured. **J** The effect of tiplaxtinin on the expression of γ-H2A.X, Bcl2, and Bax in K562 cells was analyzed by Western blotting. **K** The cytoplasmic and mitochondrial fractions of tiaplaxtinin-treated and control K562 cells were isolated, and the expression of Bax and cytochrome C (Cyto C) was analyzed by Western blotting. **L–N** K562 cells were treated with tiplaxtinin (Ti), the pan-caspase inhibitor Z-VAD-FMK (Z VAD), and the co-treatment of Ti + Z VAD. Then, the apoptosis (L), viability (M), and the activities of caspase-8 and caspase-9 (N) of the treated cells and control cells were analyzed. Data are presented as the mean ± SEM from more than 3 biological replicates. Student’s *t* test was used to estimate the statistical significance. *p < 0.05, **p < 0.01

A previous report showed that SERPINE1 protects cells from FasL/Fas-mediated apoptosis [36]. Therefore, the effect of SERPINE1 blockade on the apoptosis of CML cells was studied. The results showed that SERPINE1 silencing strongly induced apoptosis in K562 cells (Fig. 6A, Fig. S6A). Western blotting indicated that caspase-9 and PARP were activated, while the phosphorylation of STAT5 was not affected (Fig. 6B), which suggested a possible collaborative effect of the SERPINE1 inhibitor and IM on the growth of CML cells. Tiplaxtinin treatment strongly induced apoptosis in CML CD34^+^ cells but not in NBM CD34^+^ cells (Fig. 6C, Fig. S6B). Western blotting indicated that caspase-3, caspase-8, caspase-9, and PARP were all activated upon tiplaxtinin treatment (Fig. 6D). Next, the results showed that SERPINE1 silencing slightly increased the activity of caspase-8 while strongly enhanced caspase-9 activity (Fig. 6E). Similarly, caspase-9 activity was induced by tiplaxtinin in a time-dependent manner, while caspase-8 activity was induced to a much lesser extent than caspase-9 activity (Fig. 6F). As caspase-9 activation is tightly linked with internal apoptosis and mitochondrial dysfunction, the effects of tiaplaxtinin on mitochondrial activities were analyzed. The results showed that reactive oxygen species (ROS) generation was significantly enhanced by tiplaxtinin in a dose-dependent manner (Fig. 6G). The activity of mitochondria was decreased by tiplaxtinin treatment, as indicated by MitoTracker (Red CMXRos) staining (Fig. 6H, Fig. S6C). The membrane potential of mitochondria was impaired upon tiplaxtinin treatment, as shown by JC-1 staining (Fig. 6I, Fig. S6D). Western blotting showed that Bax expression was strongly increased, while Bcl-2 expression was not affected upon tiplaxtinin treatment, which strongly suggested that tiplaxtinin induced internal apoptosis. Moreover, γ-H2A.X expression was increased, suggesting the accumulation of DNA damage, possibly due to the enhancement of ROS (Fig. 6J). The analysis of protein extracts from the cytoplasm and mitochondria indicated the translocation of Bax from the mitochondria to the cytoplasm and the release of cytochrome C from the mitochondria to the cytoplasm (Fig. 6K), supporting the involvement of caspase-9-mediated internal apoptosis upon tiplaxtinin treatment. Finally, a pan-caspase inhibitor Z-VAD-FMK was used to perform rescue experiments. The co-treatment of tiplaxtinin and Z-VAD-FMK significantly decreased apoptosis and increased cell viability (Fig. 6L, M, Fig. S6E). Interestingly, Z-VAD-FMK significantly decreased the activation of caspase-9 but not caspase-8 upon tiplaxtinin (Fig. 6N), supporting the notion that caspase-9 activation plays a critical role in tiplaxtinin-induced apoptosis in CML cells.

**Fig. 7:**
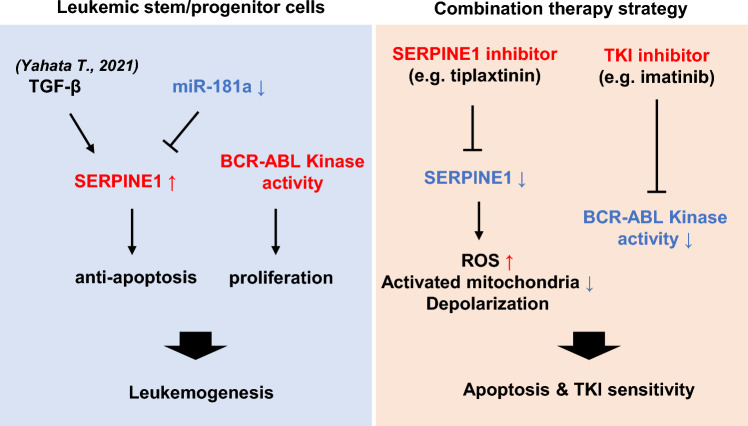
Dual targeting of SERPINE1 and BCR-ABL presents an effective approach to eradicate CML stem/progenitor cells. SERPINE1 is identified as a bona fide target of miR-181a in CML stem/progenitor cells, which highlights the importance of SERPINE1 in these cells in agreement with the previous report by Yahata T. and coworkers, who demonstrate that TGF-β induces SERPINE1 expression [46]. Importantly, the dual inhibition of SERPINE1 and BCR-ABL induces apoptosis of human CML stem/progenitor cells but not the normal control cells, which strongly promotes the strategy to improve CML treatment

Overall, we identified SERPINE1 as a bona fide target of miR-181a in CML stem/progenitor cells, which demonstrated the importance of SERPINE1 in BCR-ABL-mediated leukemogenesis. Interestingly, SERPINE1 inhibitors (e.g., tiplaxtinin) combined with TKI exhibited stronger anti-leukemia effect than a single agent, while sparing normal stem/progenitor cells (Fig. 7), which supported that the combination treatment may benefit CML management.

## Discussion

Several reports have described the tumor-suppressor role of miR-181a in CML cells [17, 28–32]; however, the effect of miR-181a on BCR-ABL-induced leukemogenesis has not yet been elucidated. In the present study, we showed that miR-181a overexpression delayed the generation of BCR-ABL-induced leukemia in mice and significantly inhibited the infiltration of leukemic cells. Additionally, the role of miR-181a in CML stem/progenitor cells has not been well studied. Herein, we showed that miR-181a inhibition enhanced the growth of NBM CD34^+^ cells and that this effect was enhanced by BCR-ABL, which strongly suggested the tumor-suppressor role of miR-181a in BCR-ABL^+^ leukemic cells. Our data also showed that miR-181a inhibited quiescent CML CD34^+^ cells and sensitized them to IM treatment. Therefore, we provided important pieces of evidence to highlight the importance of miR-181a in CML stem/progenitor cells.

The key to understanding the molecular mechanism of miR-181a in CML stem/progenitor cells is to identify the crucial target of miR-181a. Previously, some miR-181a targets were identified, including Bcl-2, RalA, and Parp1 [28, 29, 32]. In this study, we identified SERPINE1 as a bona fide target of miR-181a as our data demonstrated that miR-181a regulated the expression of SERPINE1 through a specific recognition motif. Importantly, “rescue” experiments showed that SERPINE1 played a critical role in miR-181a-mediated CML cell growth and IM response.

Interestingly, the role of SERPINE1 in leukemic stem/progenitor cells was recently reported in a mice model [46]. Similarly, we showed that Serpine1 silencing attenuated BCR-ABL-induced leukemia in a model of BaF3 cells, which agreed with the previous study. Importantly, our results showed that SERPINE1 silencing suppressed the CFC production of CD34^+^ cells from CML patients, including the quiescent subset, which expanded the previous findings.

Studies have shown that SERPINE1 inhibitors exhibit anti-leukemia effects in mice model of CML, and the combination of SERPINE1 inhibitor and TKI achieves a higher molecular response rate in CML patients than monotherapy with TKIs [47, 48]. However, the direct effect of combination therapy on CML stem/progenitor cells is still elusive. In this study, we showed that the combination of SERPINE1 inhibitor and TKI effectively suppressed the growth of IM-resistant CML CD34^+^ cells and quiescent CML CD34^+^ cells, while it had a mild effect on normal control cells. Therefore, previous studies and ours strongly promoted the combination therapy in the future.

Overall, this study has revealed a novel regulatory connection between miR-181a and SERPINE1 in CML stem/progenitor cells and provided new pieces of evidence to promote the combination therapy of SERPINE1 inhibitors and TKIs in CML treatment.

### Supplementary Information

Below is the link to the electronic supplementary material.

Supplementary file 1 (PDF 906 KB)

## Data Availability

Data supporting the findings are included in this article and the Supplemental information. The raw data and experimental materials are available from the corresponding author upon reasonable request.
