# Supplementary material for: miR-181a plays the tumor-suppressor role in chronic myeloid leukemia CD34 + cells partially via SERPINE1
Source: Cell Mol Life Sci. 2023 Dec 16;81(1):10. doi: 10.1007/s00018-023-05036-8 (PMC10725356; doi:10.1007/s00018-023-05036-8)
Supplement: Supplementary file 1 — (PDF 906 KB) [file 18_2023_5036_MOESM1_ESM.pdf]

Supplementary Information

miR-181a plays the tumor-suppressor role in chronic myeloid leukemia CD34+ cells partially via SERPINE1

Xiuyan Zhang[1,2,†], Wenjuan Ma[1], Wen Xue[1,3], Yu Wang[1,4], Pan Chen[1], Quanxue Li[5], Yuan-Yuan Li[5], Xiaohui Hu[2,6,†], Yun Zhao[1,6,7,†], Haixia Zhou[2,6,†]

[1] Cyrus Tang Medical Institute, Collaborative Innovation Center of Hematology, Soochow University, Suzhou 215123, China

[2] The First Affiliated Hospital of Soochow University, Key Laboratory of Thrombosis and Hemostasis, Ministry of Health, Suzhou 215006, China

[3] The Affiliated Nanhua Hospital, Department of Clinical Research Institute, Hengyang Medical School, University of South China, Hengyang 421002, China

[4] Jianhu Country People's Hospital, Yancheng 224700, China

[5] Shanghai-MOST Key Laboratory of Health and Disease Genomics, Shanghai Institute for Biomedical and Pharmaceutical Technologies, Shanghai 200237, China,

[6] National Clinical Research Center for Hematologic Diseases, Suzhou 215006, China

[7] MOE Engineering Center of Hematological Disease, Soochow University, Suzhou 215123, China

[†]These authors are co-corresponding authors of this work.

Table S1.

The clinical characteristics of chronic myeloid leukemia patients recruited in this study.

		CML (CP)
**No.**	Total	78
**Gender**	Female	21
	Male	53
	Missing, n(%)	4, (5.12)
**Age**	Mean	45.6
	Range	16~78
	Age<50, n(%)	41, (52.56)
	Age≥50, n(%)	33, (42.31)
	Missing, n(%)	4, (5.12)
**WBC, $\times 10^9$/L**	Mean	152.13
	Range	4.49~438
	Missing, n(%)	11, (14.10)
**Hb, g/L**	Mean	116.49
	Range	59~186
	Missing, n(%)	11, (14.10)
**Plt, $\times 10^9$/L**	Mean	377.78
	Range	61~2138
	Missing, n(%)	11, (14.10)

Note: WBC: white blood cell; Hb: hemoglobin; Plt: platelet.

Table S2.

The gene-specific primers used in this study.

Name		sequence（5'-3'）
**For RT-qPCR**		
Human Actin	F	CACCATTGGCAATGAGCGGTTCC
	R	GTAGTTTCGTGGATGCCACAGG
Human SERPINE1	F	CTCATCAGCCACTGGAAAGGCA
	R	GACTCGTGAAGTCAGCCTGAAAC
Human GATA6	F	GCCACTACCTGTGCAACGCCT
	R	CAATCCAAGCCGCCGTGATGAA
Mouse Actin	F	GAGACCTTCAACACCCCAGC
	R	ATGTCACGCACGATTTCCC
Mouse Serpine1	F	CCTCTTCCACAAGTCTGATGGC
	R	GCAGTTCCACAACGTCATACTCG
u6 snRNA	F	CGCTTCGGCAGCACATATAC
	R	TTCACGAATTTGCGTGTCATC
miR-181a	F	TGCCGAACATTCAACGCT
	R	CAGAGCAGGGTCCGAGGTA
**For SERPINE1 overexpression**		
SERPINE1	F	GGATCCATGCAGATGTCTCCAGCCCTCACC
	R	GGATCCTCAGGGTTCCATCACTTGGCCCA
**For 3'-UTR of SERPINE1**		
construct-1 (WT)	F	GAATTCAAAGGCCAGTGGAAGAAACACC
	R	CTCGAGTCTGACATTTCTTCCTCTATTCC
construct-2 (WT)	F	GAATTCGACCCCCGTCTCTTTAAAAATAT
	R	CTCGAGTACATGGCTGACGTCACCGTC
construct-1 (MT)	F	GAGTACTAGTCCCCCATCATGTGGCCCAAC
	R	GGGACTAGTACTCTGCCACCTGCAGCACCCC
construct-2 (MT)	F	AAATACTAGTAATCTAATAGAAGCCTAATCAGCC
	R	TTACTAGTATTTCACATCTGTGTGCAATTCTCC

The underlined sequences represent restriction endonuclease sites. The mutant sequences are shown in the boxes. MT, mutant.

Table S3.

The sequences of shRNA, mimics, and sponge used in this study.

name	sequence（5'-3'）
scramble	GTTCTCCGAACGTGTCACGT
shSERPINE1#1	GGAGCACGGTCAAGCAAGT
shSERPINE1#2	AGACCAACAAGTTCAACTATA
shSerpine1#1	GCTATGGGATTCAAAGTCAAT
shSerpine1 #2	ACGAAACTGGAGATGTTATAA
miR-181a mimics	AACATTCAACGCTGTCGGTGAGT
Ctrl sponge	ACTCACCGACCTATGAGTATTT
miR-181a sponge	ACTCACCGACCTATTGAATGTT

Table S4.

Antibodies used in this study.

Antibody	*Vendor*	*Catalog Number*
CD34 APC	BD	555824
CD38 PE	BD	340909
Anti-human CD32 Clone IV.3	STEMCELL Technologies	#60012
SERPINE1	Abcam	ab187262
caspase-3	Cell Signaling Technology	#9662
caspase-8	Cell Signaling Technology	#9746
caspase-9	Cell Signaling Technology	#9502
PARP	Cell Signaling Technology	#9532
STAT5	Cell Signaling Technology	#9363
pSTAT5	Cell Signaling Technology	#9359
LC3	Novus	NB100-2220
Bax	ABclonal	A19684
Bcl-2	ABclonal	A19693
COX IV	ABclonal	A11631
Cyto C	Beyotime	AC909
Tubulin	Multisciences	ab009

Figure S1

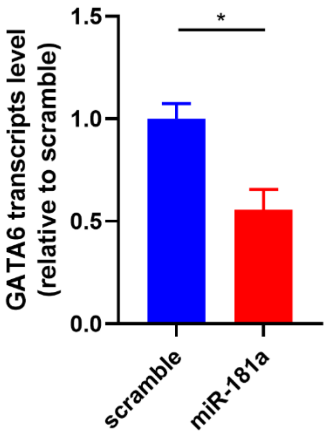

Fig. S1 GATA6 expression is inhibited by miR-181a overexpression. The expression of GATA6 in miR-181a-overexpressing and control (scramble) K562 cells was analyzed by RT-qPCR. Data are presented as the mean ± SEM. Student's *t* test was used to estimate the statistical significance. *p<0.05.

Figure S2

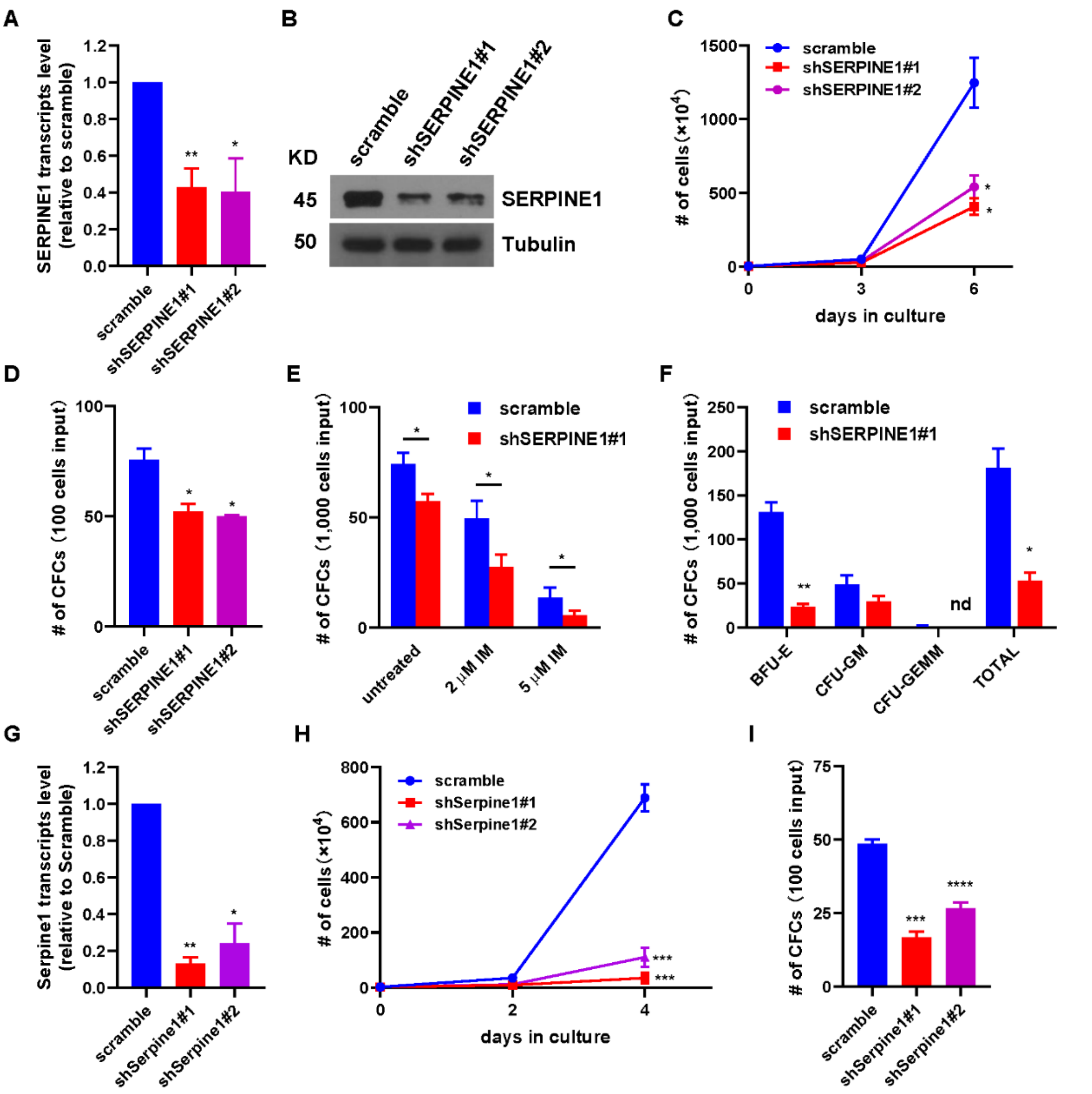

Fig. S2 Inhibition of SERPINE1 suppresses the growth of K562 and BaF3/BCR-ABL cells in vitro. **A,B** Two independent shRNA sequences against SERPINE1 and control (scramble) vectors were delivered into K562 cells, and then the transcript and protein expression of SERPINE1 was analyzed by RT-qPCR (A) and Western blotting (B). **C-E** The growth (C), colony-forming cell (CFC) abilities (D), and response to imatinib mesylate (IM) treatment (E) of SERPINE1-silenced and scramble K562 cells were assessed. **F** shSERPINE1#1 and scramble vectors were delivered into normal bone marrow (NBM) $CD34^+$ cells, and the transduced $CD34^+$ cells were purified by FACS. The CFC abilities of these cells were measured. **G-I** Two independent shRNA sequences against mouse Serpine1 and scramble vectors were delivered into BaF3/BCR-ABL cells, and then the transcript expression of Serpine1 was analyzed by RT-qPCR (G), the growth (H) and CFC abilities (I) of

Serpine1-silenced and scramble BaF3/BCR-ABL cells were analyzed. Data are presented as the mean ± SEM from more than 3 biological replicates. Student's *t* test was used to estimate the statistical significance. *$p<0.05$, **$p<0.01$, ***$p<0.001$, and ****$p<0.0001$.

Figure S3

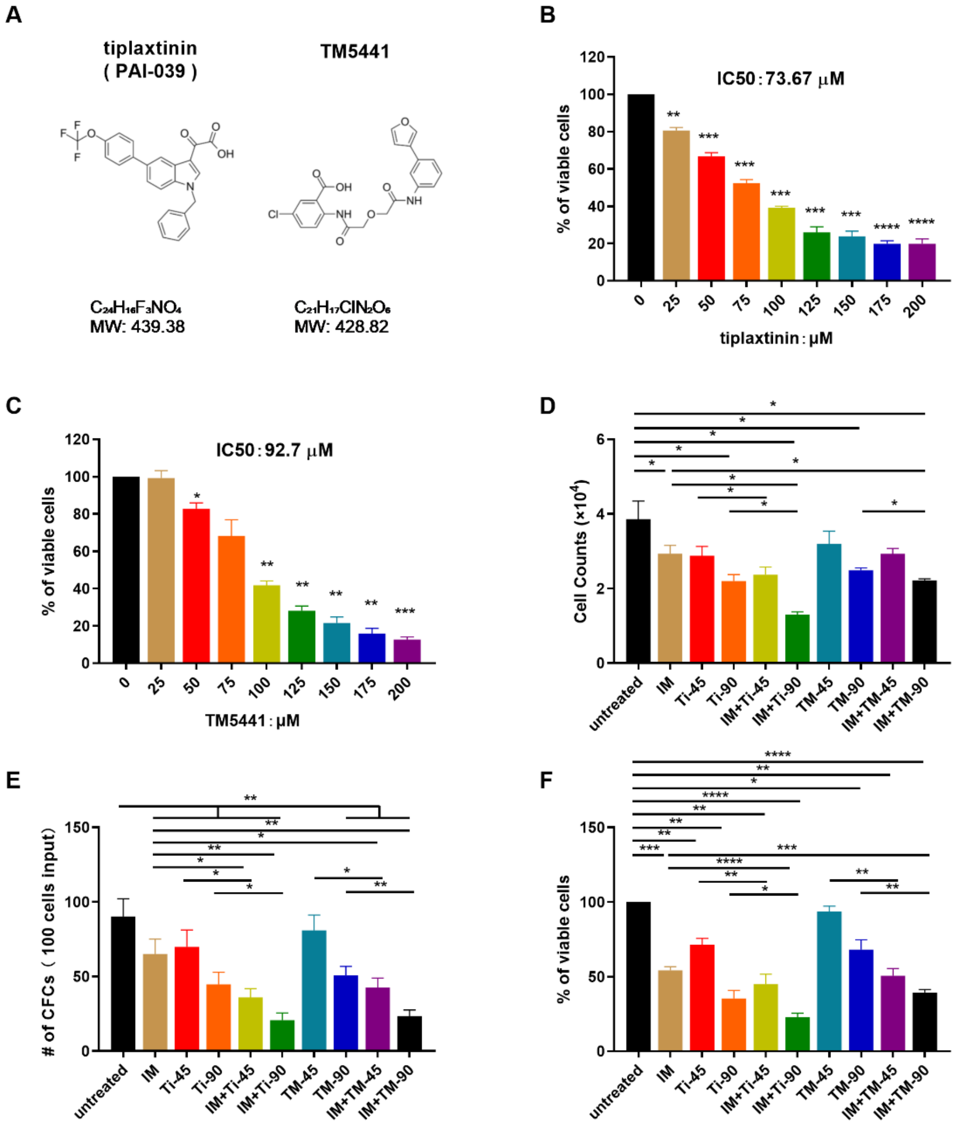

Fig. S3 The effects of SERPINE1 inhibitors on K562 and KU812 cells. **A** Two independent inhibitors against SERPINE1 are used in the present study. **B,C** KU812 cells were treated with tiplaxtinin (B) or TM5441 (C), the viability of these cells was measured by CCK-8 method. **D,E** K562 cells were treated with the combination of imatinib mesylate (IM) and tiplaxtinin (Ti) or TM5441 (TM), the cell number (D) and colony-forming cell (CFC) production (E) were measured. **F** KU812 cells were treated with the combination of IM and Ti or TM, the viability of the treated cells were measured by CCK-8 method. Data are presented as the mean ± SEM from more than 3 biological replicates. Student's *t* test was used to estimate the statistical significance. *p<0.05, **p<0.01, ***p<0.001, and ****p<0.0001.

Figure S4

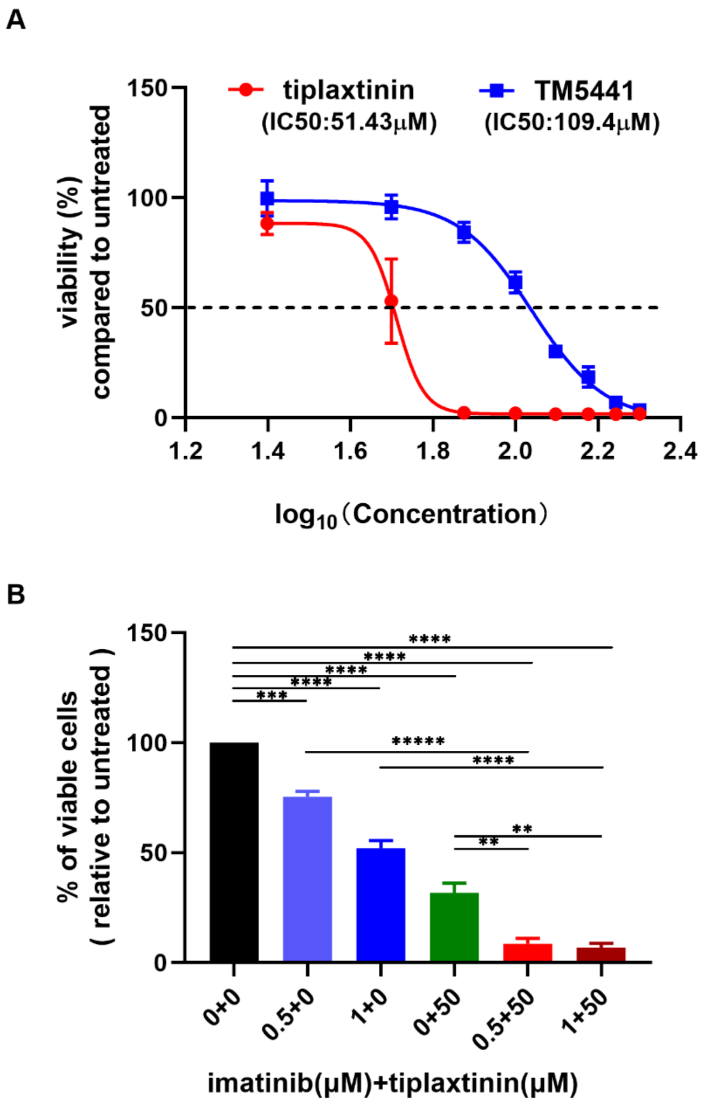

Fig. S4 The effects of SERPINE1 inhibitors on BaF3/BCR-ABL cells. **A** The viability of BaF3/BCR-ABL cells upon treatment of tiplaxtinin and TM5441 was measured. IC50 of tiplaxtinin was 51.43 μM and IC50 of TM5441 was 109.4 μM. **B** The effect of the combination of imatinib and tiplaxtinin of BaF3/BCR-ABL cells was assessed. Data are presented as the mean ± SEM from more than 3 biological replicates. Student's *t* test was used to estimate the statistical significance. **p<0.01, ***p<0.001, ****p<0.0001, and *****p<0.00001.

Figure S5

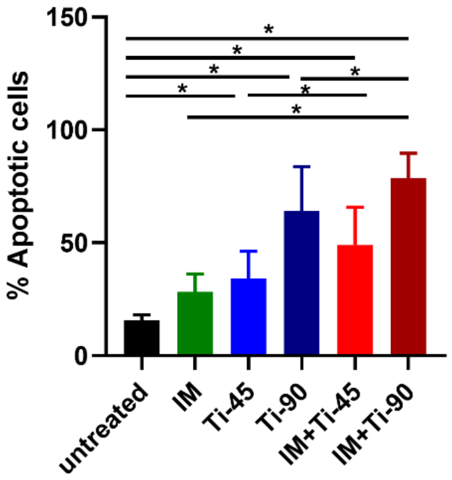

Fig. S5 The effect of the combination of imatinib and tiplaxtinin on the apoptosis of CML CD34^{+} cells. CML CD34^{+} cells were treated with the combination of imatinib mesylate (IM) and tiplaxtinin (Ti), and the apoptosis of these cells were analyzed and summarized statistically. Data are presented as the mean ± SEM from more than 3 biological replicates. Student's *t* test was used to estimate the statistical significance. *$p<0.05$.

Figure S6

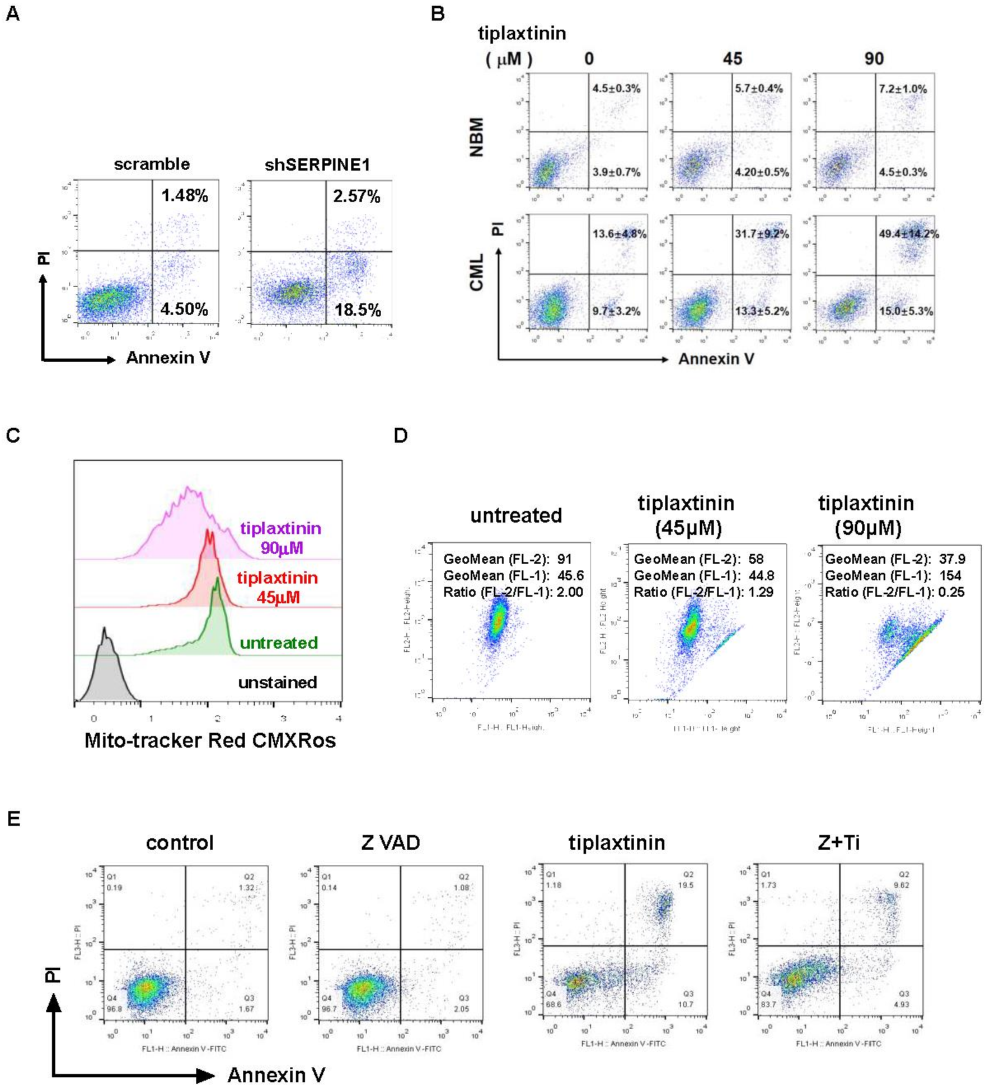

Fig. S6 Inhibition of SERPINE1 induces apoptosis. **A** SERPINE1-silenced and control (scramble) K562 cells were analyzed by Annexin V/PI staining, and the representative profiles are shown. **B** Normal bone marrow (NBM) and CML CD34$^+$ cells were treated with tiplaxtinin, and then these cells were analyzed by Annexin V/PI staining, and the representative profiles are shown. **C,D** K562 cells were treated with tiplaxtinin (45 μM and 90 μM), the treated cells and the control cells were stained with MitoTracker Red (C) and JC-1 (D). Then, the cells were analyzed by flow cytometry. The representative profiles are displayed. **E** K562 cells were treated with tiplaxtinin (Ti), Z-VAD-FMK (Z), and Ti + Z, and then these cells were analyzed with Annexin V/PI staining. The representative profiles are shown.
